# Efficacy of Topical Finasteride 0.5% vs 17α-Estradiol 0.05% in the Treatment of Postmenopausal Female Pattern Hair Loss: A Retrospective, Single-Blind Study of 119 Patients

**DOI:** 10.5826/dpc.1002a39

**Published:** 2020-04-20

**Authors:** Alfredo Rossi, Francesca Magri, Andrea D’Arino, Flavia Pigliacelli, Marta Muscianese, Pierpaolo Leoncini, Gemma Caro, Alessandro Federico, Maria Caterina Fortuna, Marta Carlesimo

**Affiliations:** 1 Division of Dermatology, Department of Internal Medicine and Medical Specialties, Sapienza University, Rome, Italy; 2 Department of Pediatric Hematology and Oncology, Istituto di Ricovero e Cura a Carattere Scientifico (IRCCS), Bambino Gesù Children’s Hospital, Rome, Italy

**Keywords:** female pattern hair loss, topical finasteride, 17α-estriadol, minoxidil

## Abstract

**Background and Objectives:**

Female pattern hair loss (FPHL) is a common form of scalp hair loss that occurs in 38% of females. Currently, minoxidil solution is the only therapy approved by the US Food and Drug Administration, but many other treatments are used, including cyproterone acetate, spironolactone, topical 17α-estradiol, and prostaglandin analogs. Systemic finasteride has been considered a treatment option in women even though its teratogenic effects tend to limit its prescription. Recently, topical finasteride has been evaluated to limit the side effect profile of the drug. The objective of the present study is to compare retrospectively the efficacy of topical 0.05% 17α-estradiol solution and a 0.5% finasteride lotion in the treatment of FPHL.

**Patients and Methods:**

We enrolled 119 postmenopausal female patients. The first group comprised 69 women treated with finasteride 0.5% and minoxidil 2%. The second group included 50 women treated with 17α-estradiol 0.05% and minoxidil 2%. At baseline and at 6- and 12- to 18-month follow-up, global photographs were systematically taken. Three operators blind to the prescribed treatment evaluated photographs using a 7-point scale. One-way analysis of variance and unpaired Student t tests were performed to analyze 7-point scale scores.

**Results:**

The improvement was statistically significant from 6 months to 12–18 months, both for finasteride (P < 0.005) and 17α-estradiol (P < 0.05). The efficacy of topical finasteride was significantly greater than that of 17α-estradiol solution, both at the 6-month (P < 0.05) and at the 12- to 18-month follow-up (P < 0.005). In general, the highest improvement was observed after 12–18 months of treatment with topical finasteride therapy.

**Conclusions:**

Topical finasteride 0.5% in combination with minoxidil 2% could represent a valid therapeutic option for the treatment of postmenopausal FPHL, showing higher efficacy than topical 17α-estradiol with minoxidil 2% both at 6-month and 12- to 18-month follow-up.

## Introduction

Female pattern hair loss (FPHL), once known as female androgenetic alopecia, is a common form of nonscarring scalp hair loss in women occurring in 38% of women between the ages of 50 and 70 years [1,2]. It is characterized by a progressive miniaturization of hair follicles especially localized in the frontal, central, and parietal regions of the scalp [3]. The pathophysiology of FPHL is still not completely clear. Even though genetic and hormonal factors are considered involved as in the more common male form [4], its link to androgen hormones is less clear as it has been shown to appear in females affected by complete androgen insensitivity syndrome [5].

The 2 drugs approved by the US Food and Drug Administration (FDA) and European Medicines Agency for the treatment of male androgenetic alopecia are topical 2% and 5% minoxidil and oral finasteride (1 mg/day) [6]. Currently, minoxidil solution is the only FDA-approved therapeutic option for FPHL. Finasteride is a selective type II 5α-reductase inhibitor, the enzyme responsible for the conversion of testosterone (T) to its more active form dihydrotestosterone (DHT). It has been considered a treatment option also in women even though its teratogenic effects tend to limit its prescription. Reports of its efficacy in women are contradictory, with less consistent results than in men [7,8]. Recently, topical formulations have been evaluated to limit the side effect profile of the drug [9]. Other commonly used therapies in FPHL are cyproterone acetate, spironolactone, flutamide, topical 17α-estradiol, mineral supplements, and prostaglandin analogs, as well as surgical therapy and light therapy [10]. The weak estrogen 17α-estradiol has been used for many years as a 0.25-mg/mL topical alternative for the treatment of both male androgenetic alopecia and FPHL given the absence of feminizing estrogenic activity [11] and its ability to weakly inhibit 5α-reductase.

The objective of the present study was to compare retrospectively the efficacy of a topical 0.05% 17α-estradiol solution and a 0.5% finasteride lotion in the treatment of FPHL.

## Methods

We performed an observational retrospective single-blind study of 119 female postmenopausal patients affected by FPHL. The purpose of this study was to evaluate and compare the efficacy of 2 different FPHL treatments: topical finasteride 0.5% and minoxidil 2% lotion (treatment 1) vs 17α-estradiol 0.05% and minoxidil 2% formulation (treatment 2).

The sample included 119 female patients affected by FPHL (I-II-III type of Ludwig classification) followed in our Department of Dermatology. Inclusion criteria and exclusion criteria for the study are summarized in Table 1.

Patients undergoing treatment 1 comprised 50 female patients, topically treated with a galenic 100-mL solution containing finasteride 0.5%, minoxidil 2%, hydrocortisone butyrate 0.08%, and cyclosilicone pentamer 16% in alcohol 96° 81.4%. Patients were between 46 and 74 years old and were observed between 2016 and 2019. Patients undergoing treatment 2 comprised 69 female patients who were prescribed a 100-mL galenic formulation containing 17α-estradiol 0.05%, minoxidil 2%, hydrocortisone butyrate 0.08%, and cyclosilicone pentamer 16% in alcohol 96° 81.87%. Patients were observed within a 10-year time range, from 2009 to 2019. The patients’ ages ranged from 45 to 79 years.

All patients were instructed to apply 1 mL of the assigned topical solution every night, on the vertex and frontal region. Follow-up visits occurred after 6 and 12–18 months. At the time of the diagnosis, and at each of the follow-up visits, pictures of the frontal and vertex scalp regions were systematically taken using a FotoFinder Video Dermatoscope (Medicam 800). The instrument was placed on the rotating arm of a head-positioning device (Canfield Scientific), permitting a standardized photograph collection.

To assess the improvement at different time intervals, 3 operators blind to the prescribed treatment evaluated photographs of the affected areas for each patient at baseline, at 6 months, and at 12–18 months from diagnosis. For the evaluation, a 7-point improvement scale was used. This is a standardized quantitative score for measuring the hair growth and clinical response: −3 = greatly decreased, −2 = moderately decreased, −1 = slightly decreased, 0 = no change, +1 = slightly increased, +2 = moderately increased, and +3 = greatly increased. All operators rated photographs for all participants at all time points, and the score was then averaged.

### Statistical Analyses

One-way analysis of variance and unpaired Student t tests were performed using SPSS software v19 (IBM, Armonk, NY, USA) and GraphPad Prism v6 (GraphPad software, San Diego, CA, USA). Confidence interval was set to 95% assuming statistically significant P values ≤ 0.05.

## Results

In our sample of 119 patients, hair loss improvement was observed in 112 women, while worsening was noticed in 7 patients. Specifically, in the group to which treatment 1 (topical finasteride) was prescribed, 48 out of 50 patients (96%) showed improvement, while 2 out of 50 showed an increase of hair loss after 6 months (both of these patients’ photographs had an average rating of −1 on the 7-point scale). In the group to which treatment 2 (17α-estradiol) was prescribed, 64 out of 69 patients (92.7%) showed improvement. Four out of 69 patients worsened after 12–18 months (average rating of −1 in all cases), while 1 patient had worse scores already at 6 months (rated −2 after 6 months and −1 after 12–18 months).

Given the small number of patients who showed a worsening of their condition following either treatment, we excluded these patients from further analyses. The following results apply only to patients who showed improvement following treatment (n = 112). We tested whether the 2 treatments led to improvements of different entity by comparing the mean ratings on the 7-point scale.

**Figure 1:**
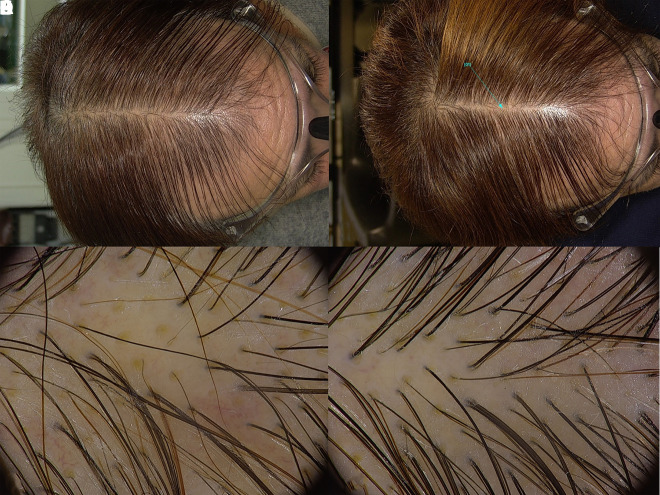
Scalp photographs and trichoscopy of a patient undergoing topical treatment with finasteride lotion, at baseline (A,C) and after 6 months of therapy (B,D).

**Figure 2:**
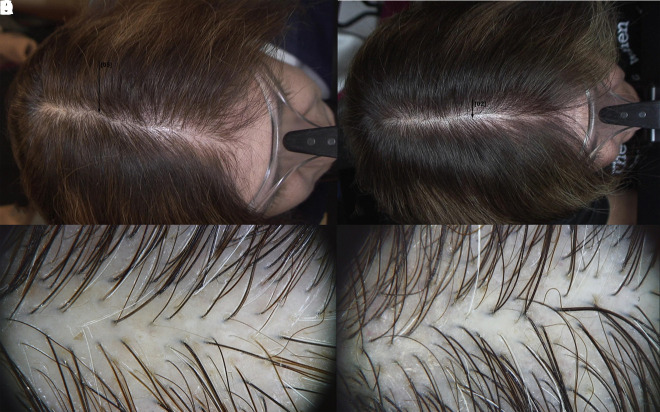
Macroscopic and trichoscopy pictures of a patient under treatment with 17α-estradiol topical lotion, at baseline (A,C) and after 6 months of therapy (B,D).

**Figure 3:**
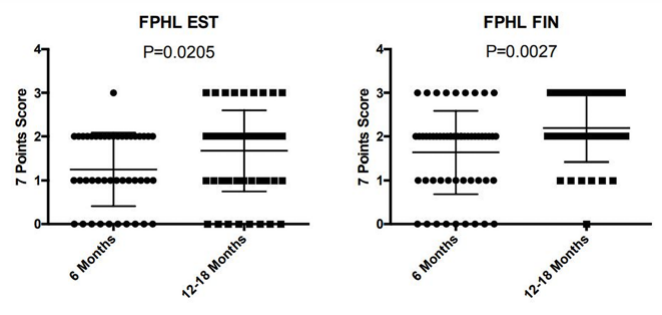
Using the 7-point scale, the improvement was statistically significant from 6 months to 12–18 months, both for finasteride (P < 0.0027) and 17α-estradiol (P < 0.0205). EST = 17α-estradiol; FIN = finasteride; FPHL = female pattern hair loss; 0 = no change; 1 = slightly increased; 2 = moderately increased; 3 = greatly increased.

**Figure 4:**
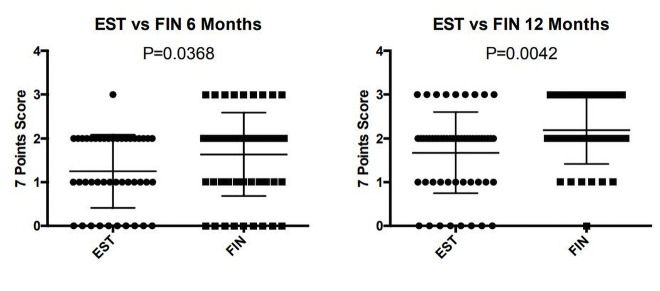
Greater efficacy of topical finasteride than 17α-estradiol solution, both at 6 months (P < 0.0368) and at 12–18 months follow-up (P < 0.0042). EST = 17α-estradiol; FIN = finasteride; 0 = no change; 1 = slightly increased; 2 = moderately increased; 3 = greatly increased.

**Figure 5:**
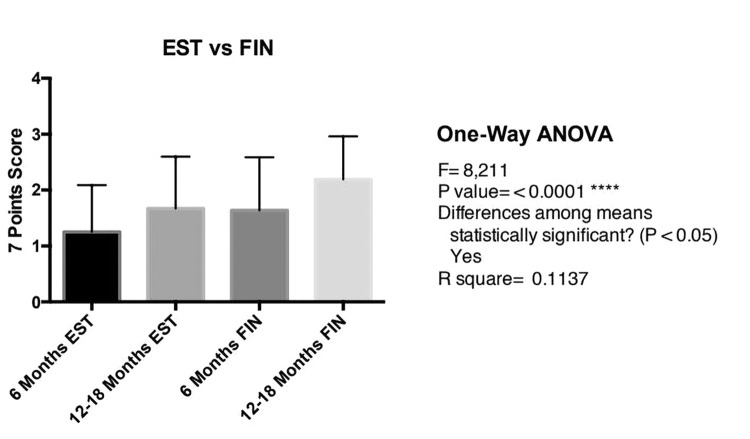
The highest improvement is observed after 12 to 18 months of treatment with topical finasteride and minoxidil therapy.

The improvement was statistically significant from 6 months to 12–18 months, both for finasteride (P < 0.005) and 17α-estradiol (P < 0.05) galenic formulations (Figures 1–3). More important, the efficacy of topical finasteride (treatment 1) was significantly greater than that of the 17α-estradiol solution (treatment 2), both at the 6-month (P < 0.05) and at the 12- to 18-month follow-up (P < 0.005) (Figure 4). In general, the highest improvement was observed after 12–18 months of treatment with topical finasteride and minoxidil therapy (Figure 5).

## Discussion

In our work, we compared 2 galenic formulations for the treatment of FPHL. The first 100-mL solution contained 17α-estradiol 0.05%, minoxidil 2%, hydrocortisone butyrate 0.08%, and cyclosilicone pentamer 16% in alcohol 96° 81.87%. The second 100-mL formulation was composed of finasteride 0.5%, minoxidil 2%, hydrocortisone butyrate 0.08%, and cyclosilicone pentamer 16% in alcohol 96° 81.4%.

Hydrocortisone butyrate is a class II steroid that blocks the inflammatory process induced by lymphocytic peri-infundibular and peristhmic infiltrate [12,13].

Minoxidil is a piperidinopyrimidine derivative and its action is believed to be due to its vasodilator properties. This is realized through the opening of potassium channels localized on the smooth muscular cells of the peripheral artery [14]. Approved in 1979 for hypertension, it is largely used as a topical product for alopecia owing to its common adverse effect of inducing hair growth. Minoxidil modifies the hair cycle, shortening the telogen phase or prolonging the anagen phase [12]. It directly stimulates the dermal papilla or the follicular hair matrix cells, causing an increase of vascular endothelial growth factor and consequently angiogenesis [12,15]. Furthermore, it has been hypothesized that it activates the prostaglandin endoperoxide synthetase enzyme type I, which induces hair growth [16].

Currently, minoxidil is the only FDA-approved therapeutic option for FPHL [17]. The 2% concentration was approved in 1991, and 5% minoxidil was approved in 2014 [18].

Systemic finasteride inhibits the type II 5α-reductase, which normally catalyzes the conversion of testosterone (T) to dihydrotestosterone (DHT), the more active form of T. Finasteride is not FDA-approved for the female population. Studies are limited and variable dosages of oral finasteride have been tested in female patients (1–5 mg/day) with conflicting results [19]. Therefore, further randomized studies are still necessary to determine the efficacy and optimal dosage of this drug. Moreover, systemic finasteride is associated with several side effects, especially sexual alterations, and its assumption is contraindicated during pregnancy and breast-feeding because of the risk of feminization of the male fetus [18].

With the aim of reducing oral finasteride side effects, a topical formulation of this drug was introduced years ago, with promising results in male patients.

Recently, Suchonwanit et al [20] studied and compared for the first time the efficacy of topical 0.25% finasteride combined with 3% minoxidil solution and 3% minoxidil solution as monotherapy in 30 FPHL postmenopausal patients. In their work, the finasteride/minoxidil solution was significantly superior to minoxidil in improvement of hair diameter.

17α-Estradiol solutions represent another therapeutic option for FPHL. This is a stereoisomer of the female hormone 17β-estradiol, which weakly inhibits 5α-reductase. Furthermore, it suppresses the 17β-dehydrogenase, causing a reductive conversion of androstenedione to T, and it stimulates the aromatase, which induces the conversion of T to estradiol [21].

In our work, through global photographs and using the 7-point rating scale, we observed that both galenic formulations determined a statistically significant improvement of hair loss from 6-month follow-up to 12- to 18-month follow-up. More important, 0.5% finasteride showed higher efficacy than 0.05% 17α-estradiol lotion, both at 6 and 12–18 months. This outcome was statistically significant.

It is interesting that finasteride-treated patients had an improvement at 6 months, which was comparable with the improvement of 17α-estradiol-treated patients at 12–18 months. Indeed, topical 17α-estradiol therapy required more time to achieve the same results of finasteride.

The greater rapidity and higher efficacy of finasteride could be explained with 2 observations. First, finasteride induces a stronger block of type II 5α-reductase, if compared with the weak inhibition of 17α-estradiol. Moreover, the inhibition of type II 5α-reductase induced by finasteride could cause a T escape, which in turn stimulates the aromatases, increasing estradiol conversion and guaranteeing an adjunctive activity similar to that of 17α-estradiol.

As we have shown, both therapies produced an improvement that was already visible at 6 months and especially after 12–18 months. Indeed, the treatment should be always continued for long periods to achieve better results.

Concerning topical finasteride’s side effects, a systemic absorption is possible, as described by Caserini et al [22]. Also, Suchonwanit et al demonstrated a lower serum level of DTH in postmenopausal women treated with it. Indeed, topical finasteride should be used only in postmenopausal women. A limit of our study was the absence of a control group. Another limit was the lack of hair analysis besides global photographs. We did not perform a computerized quantitative measure (Trichoscale) because most of our female patients refused to realize a circumscribed shaved area on the scalp with tattoos. Another limit was the subjective assessment of patients, even though all observers were blind to the therapies. Regardless, the use of the 7-point scale allowed us to perform a valid statistical analysis.

## Conclusions

Based on our results, topical finasteride 0.5% in combination with minoxidil 2% and hydrocortisone butyrate 0.08% could represent a valid therapeutic option for the treatment of postmenopausal FPHL, showing higher efficacy than topical formulation containing 17α-estradiol 0.05% in combination with minoxidil 2% and hydrocortisone butyrate 0.08%, both at 6-month and 12- to 18-month follow-up.

## Figures and Tables

**Table 1:** Inclusion and Exclusion Criteria for the Study

Inclusion criteria:
Postmenopausal patients Female pattern hair loss Type I-II-III of Ludwig classification No other concomitant treatments, including hormonal replacement therapy
**Exclusion criteria:**
Concomitant treatments of the scalp Associated systemic diseases and/or systemic therapies which could influence hair growth (such as oncological patients undergoing chemotherapy) Concomitant scalp diseases (such as lichen planopilaris, alopecia areata, frontal fibrosing alopecia, telogen effluvium)

## References

[1] Birch MP, Messenger JF, Messenger AG (2001). Hair density, hair diameter and the prevalence of female pattern hair loss. Br J Dermatol.

[2] Price VH (2003). Androgenetic alopecia in women. J Investig Dermatol Symp Proc.

[3] Olsen EA (2005). Female pattern hair loss and its relationship to permanent/cicatricial alopecia: a new perspective. J Investig Dermatol Symp Proc.

[4] Messenger AG (2011). Hair through the female life cycle. Br J Dermatol.

[5] Cousen P, Messenger A (2010). Female pattern hair loss in complete androgen insensitivity syndrome. Br J Dermatol.

[6] Rossi A, Anzalone A, Fortuna MC (2016). Multi-therapies in androgenetic alopecia: review and clinical experiences. Dermatol Ther.

[7] Iorizzo M, Vincenzi C, Voudouris S, Piraccini BM, Tosti A (2006). Finasteride treatment of female pattern hair loss. Arch Dermatol.

[8] Olszewska M, Rudnicka L (2005). Effective treatment of female androgenic alopecia with dutasteride. J Drugs Dermatol.

[9] Lee SW, Juhasz M, Mobasher P, Ekelem C, Mesinkovska NA (2018). A systematic review of topical finasteride in the treatment of androgenetic alopecia in men and women. J Drugs Dermatol.

[10] Choe SJ, Lee S, Choi J, Lee WS (2017). Therapeutic efficacy of a combination therapy of topical 17alpha-estradiol and topical minoxidil on female pattern hair loss: a noncomparative, retrospective evaluation. Ann Dermatol.

[11] Moos WH, Dykens JA, Howell N (2008). 17α-Estradiol: a less-feminizing estrogen. Drug Dev Res.

[12] Rossi A, Iorio A, Scarnò M (2014). Use of topical minoxidil, 17α-estradiol and hydrocortisone butyrate in frontal fibrosing alopecia. Eur J Inflamm.

[13] Whiting DA (1993). Diagnostic and predictive value of horizontal sections of scalp biopsy specimens in male pattern androgenetic alopecia. J Am Acad Dermatol.

[14] Rossi A, Cantisani C, Melis L, Iorio A, Scali E, Calvieri S (2012). Minoxidil use in dermatology, side effects and recent patents. Recent Pat Inflamm Allergy Drug Discov.

[15] Walsh DS, Dunn CL, James WD (1995). Improvement in androgenetic alopecia (stage V) using topical minoxidil in a retinoid vehicle and oral finasteride. Arch Dermatol.

[16] Buhl AE, Waldon DJ, Conrad SJ (1992). Potassium channel conductance: a mechanism affecting hair growth both in vitro and in vivo. J Invest Dermatol.

[17] Rogers NE, Avram MR (2008). Medical treatments for male and female pattern hair loss. J Am Acad Dermatol.

[18] Fabbrocini G, Cantelli M, Masara A, Annunziata MC, Marasca C, Cacciapuoti S (2018). Female pattern hair loss: a clinical, pathophysiologic, and therapeutic review. Int J Womens Dermatol.

[19] Hu AC, Chapman LW, Mesinkovska NA (2019). The efficacy and use of finasteride in women: a systematic review. Int J Dermatol.

[20] Suchonwanit P, Iamsumang W, Rojhirunsakool S (2019). Efficacy of topical combination of 0.25% finasteride and 3% minoxidil versus 3% minoxidil solution in female pattern hair loss: a randomized, double-blind, controlled study. Am J Clin Dermatol.

[21] Kim JH, Lee SY, Lee HJ, Yoon NY, Lee WS (2012). The efficacy and safety of 17α-estradiol (Ell-Cranell® alpha 0.025%) solution on female pattern hair loss: single center, open-label, non-comparative, phase IV study. Ann Dermatol.

[22] Caserini M, Radicioni M, Leuratti C, Annoni O, Palmieri R (2014). A novel finasteride 0.25% topical solution for androgenetic alopecia: pharmacokinetics and effects on plasma androgen levels in healthy male volunteers. Int J Clin Pharmacol Ther.

